# A dual-channel endoscope for quantitative imaging, monitoring, and triggering of doxorubicin release from liposomes in living mice

**DOI:** 10.1038/s41598-017-15790-y

**Published:** 2017-11-14

**Authors:** Jeremy Kress, Daniel J. Rohrbach, Kevin A. Carter, Dandan Luo, Chien Poon, Semra Aygun-Sunar, Shuai Shao, Shashikant Lele, Jonathan F. Lovell, Ulas Sunar

**Affiliations:** 1 Department of Biomedical, Industrial & Human Factors Engineering, Wright State University, Dayton, OH USA; 2 Department of Biomedical Engineering, University at Buffalo, Buffalo, NY USA; 3 Department of Pharmacology & Toxicology, Wright State University, Dayton, OH USA; 4 Department of Medicine, Roswell Park Cancer Institute, Buffalo, NY USA

## Abstract

Doxorubicin (Dox) is approved for use in liposomal form for the treatment of ovarian cancer. We previously developed a long-circulating Dox formulation in liposomes containing small amounts of porphyrin-phospholipid, which enables on-demand drug release with near-infrared irradiation. In this study, we present and evaluate a dual-modal, dual-channel light endoscope that allows quantitative reflectance and fluorescence imaging for monitoring of local Dox concentrations in target areas. The endoscope consists of two flexible imaging fibers; one to transmit diagnostic and therapeutic light to the target, and the other to detect fluorescent and reflected light. Thus, the endoscope serves for imaging, for light delivery to trigger drug release, and for monitoring drug concentration kinetics during drug release. We characterized the performance of this endoscope in tissue phantoms and in an *in vivo* model of ovarian cancer. This study demonstrates the feasibility of non-invasive, quantitative mapping of Dox distribution *in vivo* via endoscopic imaging.

## Introduction

A major challenge in the treatment of advanced ovarian cancer is the presence of disseminated microscopic tumor nodules in the intraperitoneal cavity. Despite surgery and adjuvant chemotherapy, as many as 50% of patients can show occult disseminated disease^1^. Recent efforts have aimed at improving detection and treatment of these small nodules, also termed micrometastases (micromets)^2–6^. Conventional imaging techniques, such as computed tomography (CT), magnetic resonance imaging (MRI), positron emission tomography (PET), and ultrasound, demonstrate less sensitive detection than reassessment surgeries^1,7–9^. Furthermore, treatment via systemic chemotherapy can have toxic side effects, decreasing the patient’s quality of life^10^. Liposomal nanocarriers have been developed to enhance the biodistribution and efficacy of anti-cancer drugs including doxorubicin (Dox)^11–13^. Liposomal Dox is currently used in patients with recurrent ovarian carcinoma^14^. While liposomal formulations decrease some side effects, drug delivery is hampered by physiological barriers and release kinetics, so that biodistribution and bioavailability at the desired site are sub-optimal^15^. Significant research efforts have sought better ways to release cargo from liposomes in the target area only. Some methods rely on intrinsic tumor properties, such as pH differences^16^ or enzymes^17,18^, while others rely on external mechanisms. For example, externally triggered heat release of drugs from liposomes has progressed for years^19,20^; drug release occurs when surrounding temperatures are raised a few degrees above body temperature via direct or indirect heating^21,22^. Such mechanisms are not optimal for triggered release and the narrow thermal operating window precludes carrier stability at physiological temperatures. The combination of chemo- and photo-therapy is also being explored^23^. To improve biodistribution and bioavailability, liposomes can be triggered with light to release their cargos^24^. We have recently developed porphyrin-phospholipid (PoP) liposomes that can be permeabilized on demand with near infrared (NIR) light to release entrapped drugs with excellent temporal and spatial control^25–29^. A formulation of long-circulating doxorubicin in PoP liposomes (LC-Dox-PoP) was developed that enables tumor ablation with similar long circulation and expected systemic efficacy and toxicity as DOXIL®, although its toxicity has not yet been thoroughly assessed^30^.

To rationally develop an image-guided approach to local delivery of chemotherapies, knowledge about the concentration of Dox at the target site is essential. Since Dox fluoresces, fluorescence spectroscopy or imaging can be implemented for quantification of Dox content *in vivo* ^31,32^. In optically clear media, the drug’s fluorescence is directly proportional its concentration, and there is no attenuation of treatment light. Thus, the fluence rate and time will directly determine the total light dose. However, the raw fluorescence signal *in vivo* is known to be strongly affected by tissue optical absorption and scattering properties, and thus is not directly related to the true Dox concentration. In addition, the background optical properties attenuate the treatment light, potentially shielding the liposomes and slowing drug release.

Several spectroscopic methods have been utilized for quantification of drug concentrations *in vivo* ^31–39^. Although spectroscopic methods are useful, they can only provide average values from point measurements and cannot provide drug distributions. We have shown successful quantification of porphyrin and Dox concentration maps by using wide-field spatial frequency domain imaging (SFDI) technique^40,41^. SFDI is a relatively new method that allows fast, quantitative imaging of tissue optical absorption and scattering parameters, as well as fluorescence concentrations of administered drugs^42–45^. Spatially modulated light (structured illumination) is projected onto tissue and the tissue’s spatially modulated transfer function is measured, which is related to optical absorption and scattering^42^. Recently, the SFDI technique has been expanded to include endoscopic applications to obtain optical property maps^46–48^. Here, we combined endoscopic spatial frequency domain imaging (eSFDI) with quantitative fluorescence imaging. Our dual-channel endoscopic approach uses two, flexible image fibers for patterned illumination and detection in both reflectance and fluorescence imaging modes to quantify optical and fluorescence parameters, respectively. SFDI-quantified optical parameters provide *a priori* information for a correction factor to obtain accurate drug fluorescence concentrations. This approach allows quantification of absolute Dox fluorescence concentration by compensating for variations in fluorescence signal due to absorption and scattering at both excitation and emission wavelengths^40,43,49^. The endoscope also works in dual mode to provide a unique combined platform for imaging plus treatment light delivery. In the delivery mode, the endoscope channel can project an optimized treatment field onto micromets for optimal drug delivery. This endoscope is based on mesoscopic diffuse optical imaging, having scales of approximately ten to hundreds of microns of resolution and a few millimeters of penetration depths^50^. It works both in reflectance and fluorescence imaging modes. Since eSFDI approach is non-contact, wide-field and fast, it can determine pharmacokinetics of drug release in near real-time by utilizing a highly sensitive fluorescence contrast. Laminar optical tomography (LOT) is another mesoscopic diffuse optical imaging technique like SFDI that can also provide fluorescence contrast^51–54^. However, LOT is scanning-based and cannot quantify optical parameters and absolute drug fluorescence concentration.

In this work, we report the development, characterization, and application of a novel endoscope based on SFDI technique. We explored the accuracy of quantification in both tissue-simulating phantoms and an *in vivo* mouse model for ovarian cancer. We then investigated the time-dependent release kinetics with respect to light energy to ensure full release of the liposomal cargo. Our results support the feasibility of improving localized drug delivery to tumor tissues through a dual-modal eSFDI platform, which quantifies drug release in near real-time so that treatment light delivery can be continuously monitored.

## Results

### System Calibration

**Figure 1:**
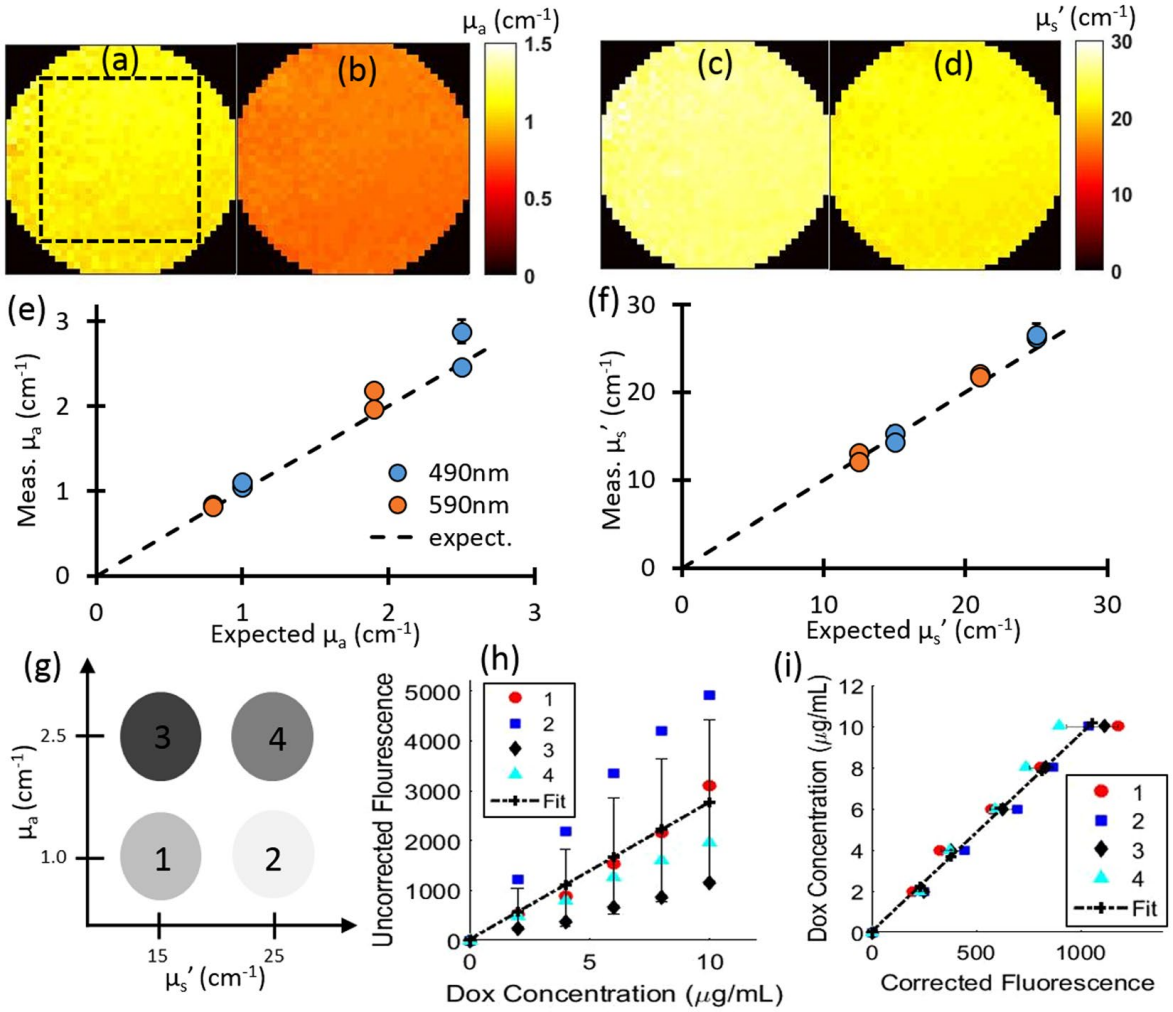
System calibration and optical property correction. (**a**) and (**b**) show absorption maps of one calibration phantom at 490 nm and 590 nm respectively. The dashed square indicates the ROI. (**c**) and (**d**) show scattering maps of the same calibration phantom at 490 nm and 590 nm respectively. (**e**) and (**f**) show the quantification of absorption and scattering all four calibration phantoms respectively. (**g**) A schematic of the acquired phantom optical properties. (**h**) Uncorrected (raw) Dox fluorescence vs. Dox concentration, showing the large variation with respect to background optical property differences. (**i**) Attenuation-corrected Dox fluorescence vs. Dox concentration using all phantoms at different absorption and scattering parameters, indicating a strong linear relationship with respect to Dox concentration. Error bars represent standard deviation between phantoms.

We characterized the system with tissue-simulating reflectance and fluorescence phantoms formulated with varied, but known, optical properties and Dox concentrations. Phantoms were prepared using Intralipid 20% (Fresenius Kabi) for scattering and India Ink (Higgins) for absorption. Four phantoms were prepared with different combinations of optical properties: µ_a_ = 1.0 and 2.5 cm^−1^, µ_s_’ = 15.0 and 25.0 cm^−1^ at 490 nm and µ_a_ = 0.8 and 1.9 cm^−1^, µ_s_’ = 12.5 and 21.0 cm^−1^ at 590 nm. These wavelengths correspond to the excitation and emission wavelengths for Dox. Each phantom had a total volume of 100 mL. The optical properties at each wavelength were quantified by using SFDI. Figure 1a–d show the optical property maps obtained from one of the four calibration phantoms. The images are circularly cropped to avoid the far edges of the endoscope to provide a better visualization of the variations in optical property images. Figure 1e and f show the quantification of optical properties for each of the four phantoms. The average errors in quantifying absorption and scattering were 6.9% and 4.4% respectively. Errors were typically under 10%, except for the phantom with the highest absorption and scattering case (2.5 cm^−1^ and 25 cm^−1^ at 490 nm respectively). For this case, the errors in absorption quantification at 490 nm and 590 nm were 15.2% and 15.1%, respectively. Errors in the quantified scattering were always under 10%.

For the calibration of Dox concentration, a stock solution of 1.0 mg/mL free-Dox was used. After baseline eSFDI reflectance and fluorescence measurements were taken for each phantom, increasing volumes of free-Dox were added to each (0, 200, 400, 600, 800, and 1000 µL) and fluorescence measurements acquired at each addition. Reconstructed raw fluorescence values with respect to concentration were used to obtain a calibration curve for conversion to absolute Dox concentrations. Mean and standard deviation values of intensity count for each region of interest (ROI) were plotted with respect to the concentration as shown in Fig. 1.

We chose a 150 × 150 pixel-sized ROI (total resolution 250 × 250 pixels at 4 × 4 binning) for the target area, which was obtained from the threshold determined by the intensity counts values equal or above the 50% of the peak intensity. As Fig. 1h clearly indicates, the raw fluorescence signal showed substantial variations with respect to optical parameters, but attenuation-corrected fluorescence showed substantially less variation with respect to Dox concentrations (Fig. 1i). The uncorrected raw fluorescence signals had a poor correlation with the Dox concentration (r^2^ = 0.56) and a high average variation ~67%, which improved substantially with the corrected fluorescence signal (r^2^ = 0.99) and a lower average variation of ~10%. The larger variation at higher concentration (~15%) is likely due to the increased error in absorption quantification for the high absorption and scattering phantom.

### Long-circulating Dox in PoP Liposomes

**Figure 2:**
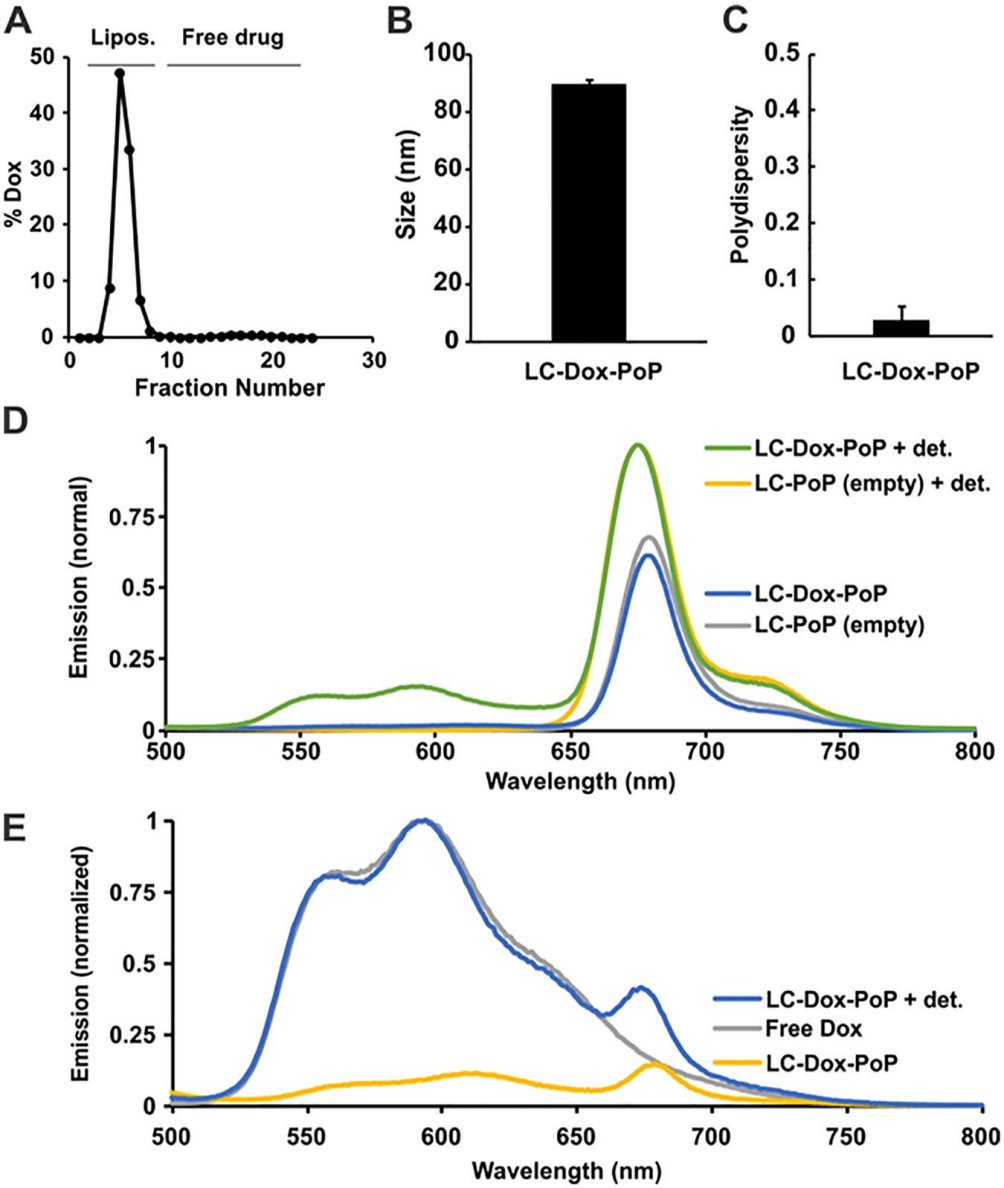
LC-Dox-PoP characteristics. (**A**) Gel filtration showing active Dox loading in liposomes (over 95% efficient). (**B**) LC-Dox-PoP size measured by dynamic light scattering. (**C**) LC-Dox-PoP polydispersity. (**D**) PoP emission spectra of LC-Dox-PoP or empty PoP liposomes (without Dox) with or without detergent disruption with 0.25% Triton X-100 (“+det”). Dilute samples were measured in phosphate-buffered saline with 420 nm excitation. (**E**) Dox emission spectra of indicated samples measured in phosphate-buffered saline with 480 nm excitation.

Loading efficiency of Dox into LC-Dox-PoPs was over 95%, (Fig. 2A). The liposome diameter was around 85 nm (Fig. 2B) and a low polydispersity index of less than 0.1 was observed (Fig. 2C). As shown in Fig. 2D, LC-Dox-PoPs, and the analogous unloaded, empty liposomes (LC-PoPs), exhibit near-infrared emission based on the presence of PoP. The molar loading, which is just 2%, results in some, but less than 50% fluorescence self-quenching of PoPs. In contrast, the Dox spectra, as shown in Fig. 2E, shows that substantial fluorescence self-quenching is observed in the intact LC-Dox-PoP. This is due to Dox forming sulfate crystals and aggregates in the liposomal core. These crystals and aggregates have diminished fluorescence compared to the fully solubilized drug.

### Reducing Distance/Height Variation with Surface Profilometry

Since the surface of an imaged object might not be flat, the distance (height) between the endoscope tip and the object can show variations. To quantify the effects of these variations on optical parameters, we performed phantom experiments in which we changed the distance by using a micro-positioner and calibrated the endoscope imaging system accordingly. Results of the phase profilometry phantom calibration are shown below. The calibration was performed at fixed heights below a reference frame [0, 2, 4, 6, and 8 mm].

**Figure 3:**
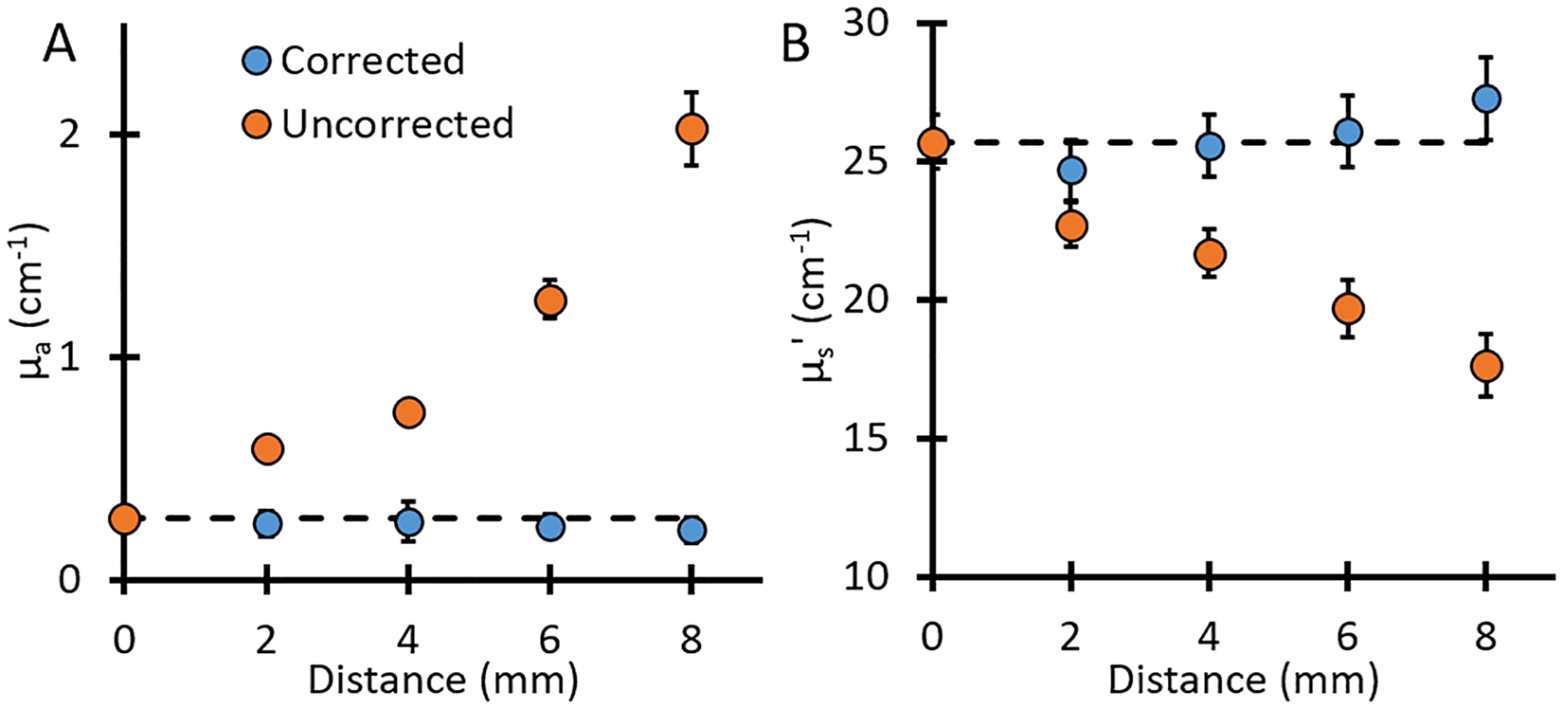
Optical property correction for distance variation. The quantification of (**A**) absorption and (**B**) reduced scattering parameters at different heights using SFDI vs. height-corrected SFDI. Dashed line in each shows the expected value. Error bars represent the standard deviation of the region of interest.

As Fig. 3A shows, changes in the working distance between the endoscope and the object had a large effect on the quantification of absorption parameter. For example, at 8 mm distance, the farthest distance tested, the uncorrected SFDI resulted in absorption parameter values 7x higher than expected, with a percent error of ~630%, while a 31% error in the reduced scattering parameter was observed (Fig. 3B). Using the distance correction algorithm, the error at 8 mm was only ~19% for the absorption parameter and ~6% for the reduced scattering parameter. The correction algorithm also produced more consistent results and less variation as a function of distance, with an average error of only ~9% for the absorption parameter and ~2% for the reduced scattering parameter.

**Figure 4:**
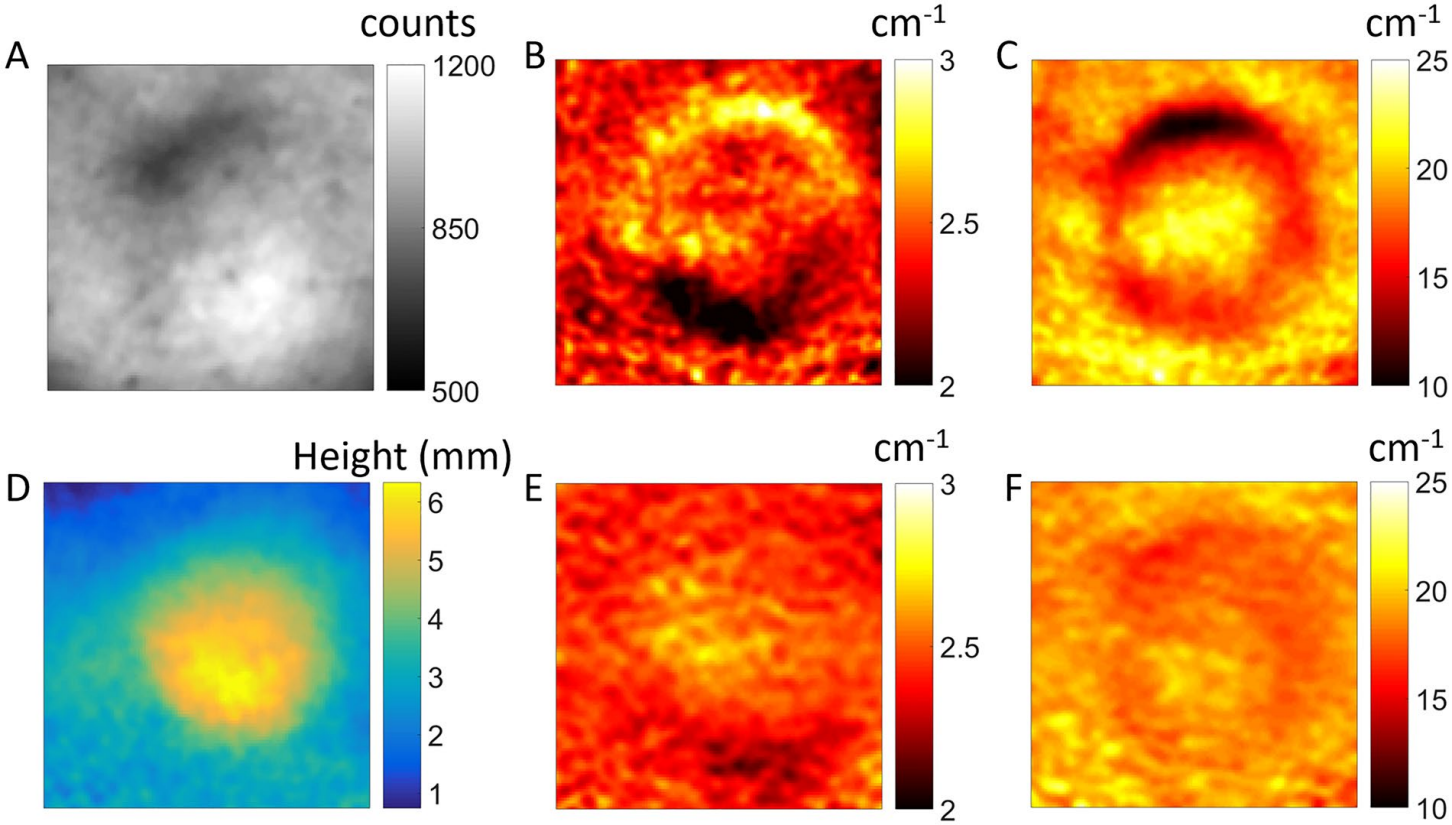
Surface profile correction example on a mouse tumor. (**A**–**C**) Shows the white light structural image of mouse skin surface with tumor mass (raw minus dark), absorption, and reduced scattering, respectively. (**D**–**F**) Shows the phase map (at AC 2.0 cm^−1^ spatial frequency), height-corrected absorption, and height-corrected reduced scattering, respectively.

Figure 4A shows a reflectance image of a mouse tumor. It is clear that there are notable height and illumination variations (brighter area on the bottom of the tumor, darker area on the top) that translate into variations in optical property quantification for the absorption parameter (Fig. 4B) and the reduced scattering parameter (Fig. 4C). After implementing surface profilometry to calculate the height variations on the mouse (Fig. 4D), and applying the height correction to SFDI quantification, the corrected absorption and scattering maps (Fig. 4E and F, respectively) showed greatly reduced height and illumination variations.

### Dox release quantification in phantoms by eSFDI

**Figure 5:**
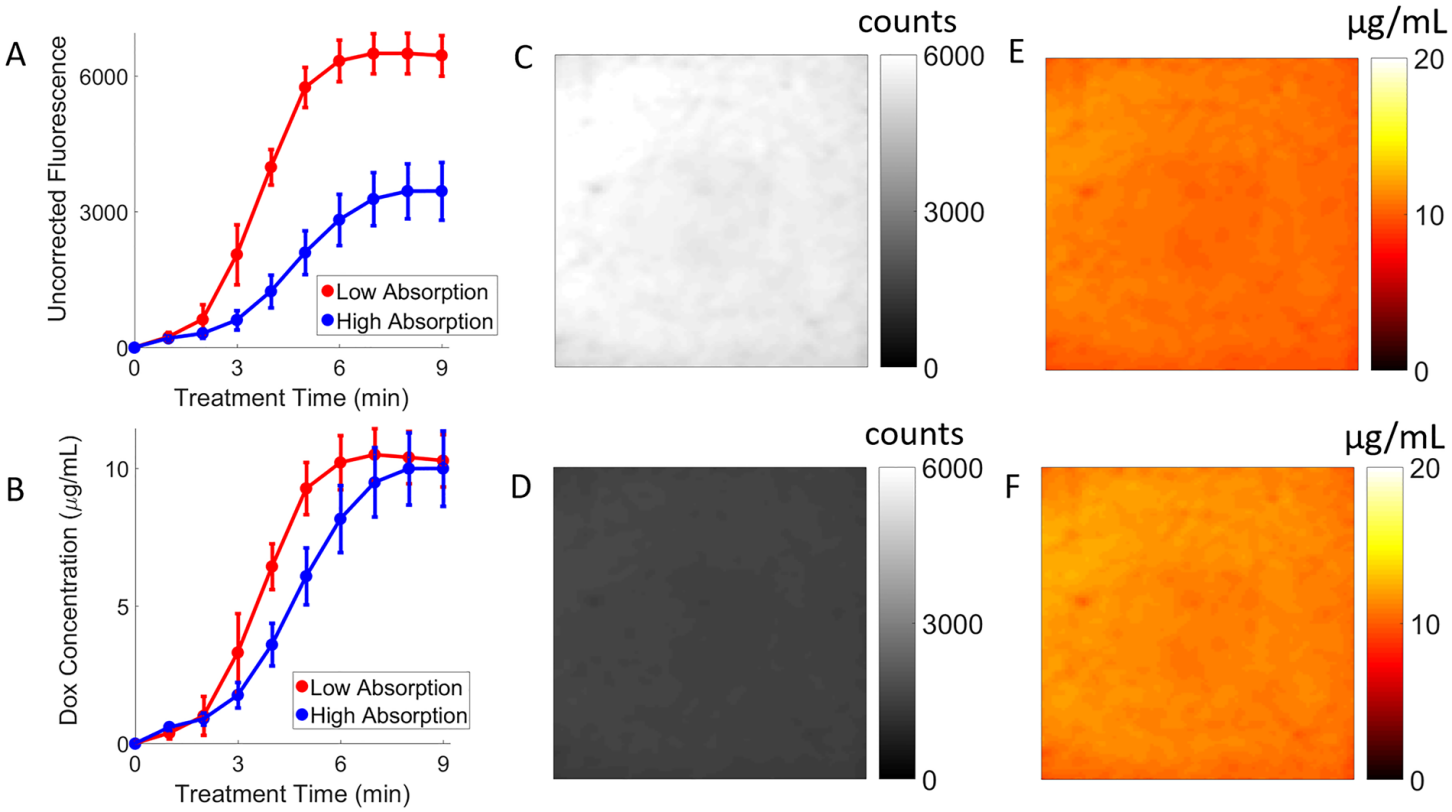
LC-Dox-PoP release kinetics in phantoms. (**A**) Dox release as characterized by uncorrected raw fluorescence changes. (**B**) Dox release as characterized by corrected, quantified Dox fluorescence concentration changes. Error bars represent standard deviation in Dox concentration for the given ROI. The uncorrected images for the low (**C**) and high (**D**) absorbing phantoms show a large signal difference after release. The corrected Dox fluorescence images for the low-absorbing (**E**) and high-absorbing phantoms (**F**) show much less variation.

Figure 5A shows the Dox release curve based on the raw fluorescence signal, indicating a large difference in the final values for Dox fluorescence signals. This is most likely because the raw fluorescence signal is strongly affected by the optical parameters. The high absorption sample shows a much lower release of Dox content. However, correction for the effects of background optical parameters reveals that both phantoms did release similar concentrations of Dox (Fig. 5B). The release time for the high-absorbing phantom was longer than for the low-absorbing phantom (8 min versus 6 min, respectively). The delay in the release kinetics (longer light dose required) for the high-absorbing phantom is expected, since the treatment light is more attenuated due to higher background absorption. As expected, the release kinetic characteristics are strongly dependent on the delivered light fluence rate and energy.

Figure 5C–F show representative images of phantoms with the same initial amount of LC-Dox-PoP content (1000 μL of 1 mg/mL drug added to 100 mL phantom, equivalent to 10.0 µg/mL Dox concentration), but with different optical absorption parameters (µ_a_). The first phantom (Fig. 5C) is at µ_a_ = 0.5 cm^−1^, while the second phantom (Fig. 5D) is at µ_a_ = 1.0 cm^−1^. The scattering parameter was at the same value for both phantoms (µ’_s_ = 20 cm^−1^).

The uncorrected fluorescence images (Fig. 5C–D) show a higher Dox fluorescence signal for the low-absorbing phantom compared to the high-absorbing one (5055 ± 101 a.u. vs. 1149 ± 54 a.u., respectively), due to decreased light attenuation in the low-absorbing phantom. Hence the image contrast was lower in the high-absorbing phantom. However, the corrected Dox concentration maps (Fig. 5E–F) show that both phantoms have a similar Dox fluorescence concentration, close to the actual known Dox concentration value (9.8 ± 0.6 µg/mL and 10.3 ± 0.7 µg/mL, corresponding to 2.0% and 3.0% error, respectively).

### Dox release quantification in mouse by eSFDI

**Figure 6:**
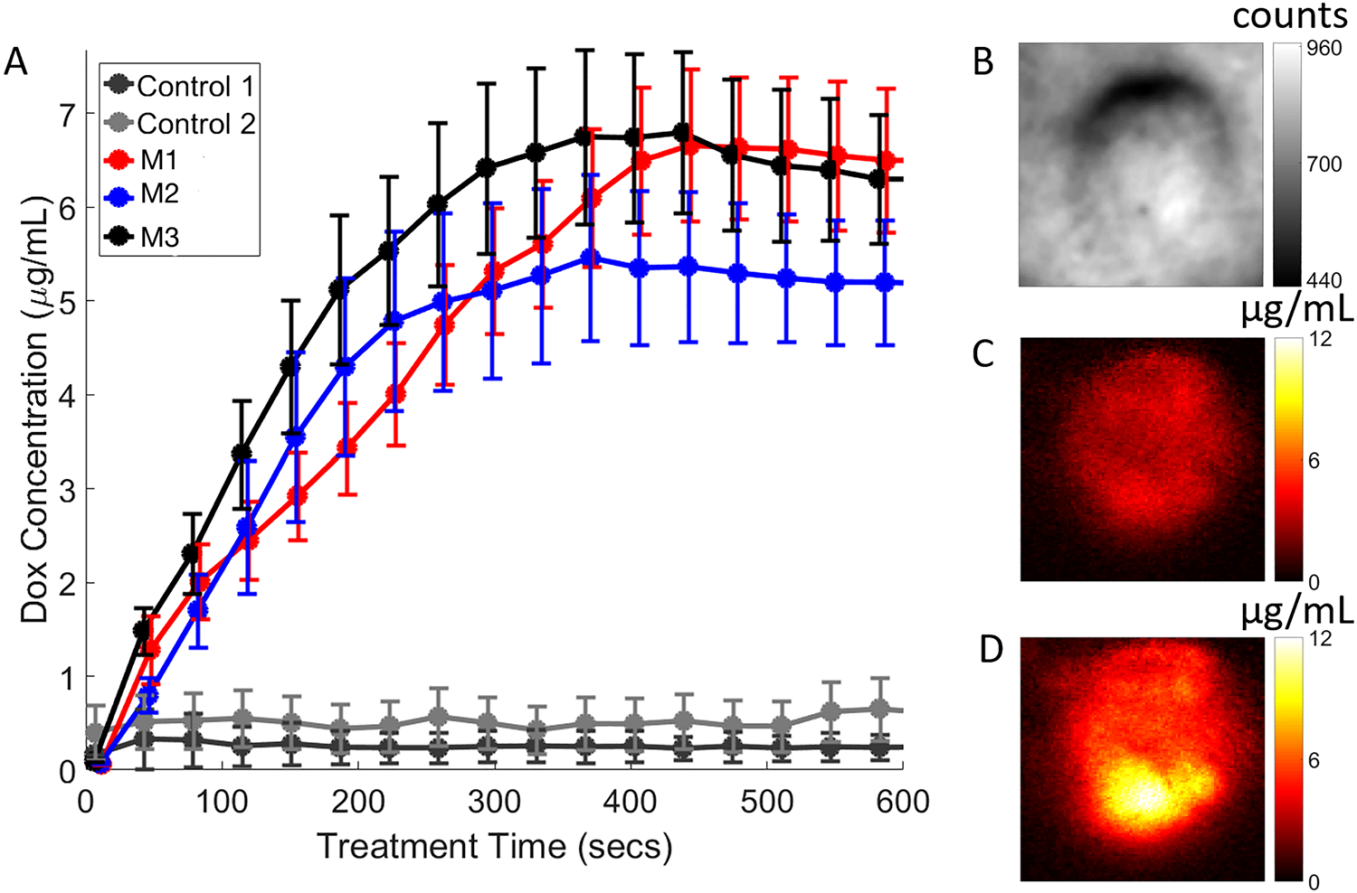
Quantified drug release in mice. (**A**) *In vivo* release kinetics curves with respect to irradiated light treatment time. (**B**) A representative white light structural image related to mouse 3 (M3), showing the Dox release in the tumor. (**C**) Quantified Dox fluorescence concentration map 1-minute post treatment light irradiation. (**D**) Quantified Dox fluorescence concentration map 10-minute post treatment light irradiation. The video, Exp 3.avi, shows the release kinetics, changes in released drug concentration with respect to treatment light irradiation for M3.

For all treated mice (N = 9), the average of quantified Dox concentration was 1.0 ± 0.2 µg/mL before release and 8.0 ± 3.0 µg/mL at final release. From the release kinetics curves, we determined the average time to reach 95% of the full release as 423 ± 129 seconds, with the quickest release as 288 seconds and the longest as 600 seconds. For visualization purposes, we show representative Dox release curves from 5 mice, 3 of the 9 treated plus 2 control mice in Fig. 6, where Control 1 corresponds to no drug injected, but treatment light applied. Control 2 corresponds to drug injected, but no treatment light applied. The quantified drug release kinetics (Fig. 6A) is shown at the tumor site on the mouse flank whose reflectance image is shown in Fig. 6B. It is clear from the release curves that time-dependent release kinetics exhibit some variations with respect to irradiated treatment time (delivered light energy). For example, mouse-1 (M1) showed slower release kinetics than mouse-3 (M3). For all the release kinetics curves, the ROIs were taken at 50% threshold of peak release value, which was generally near the center of the tumor. Figure 6C and D show the representative images of the quantified Dox fluorescence concentration maps obtained from mouse-3 (M3) at 1-minute post, and 10-minute post treatment light irradiations, respectively.

### *Ex vivo* results

**Figure 7:**
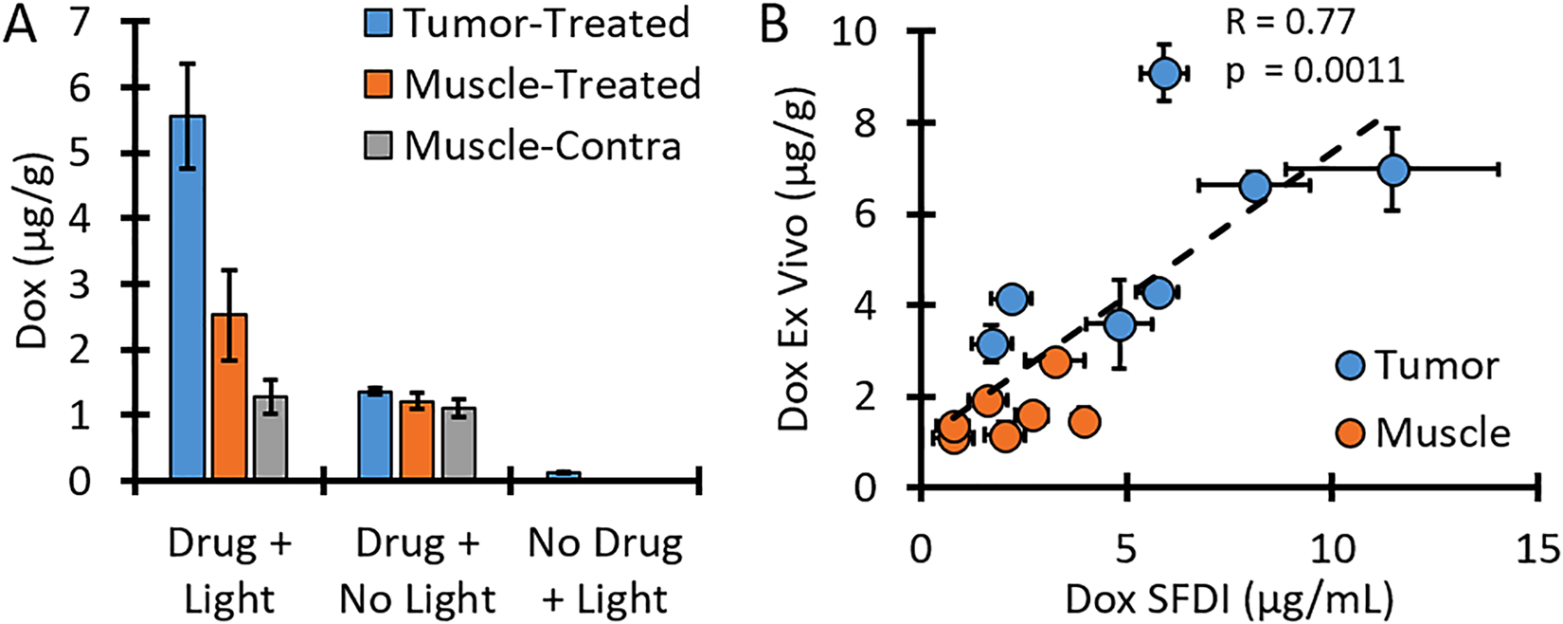
*Ex vivo* Dox quantification. (**A**) The average Dox concentration for each tissue type from all mice. Tumor and Muscle-Treated received treatment light while Muscle-Contra did not receive any light. (**B**) Relationship between Dox concentrations quantified with eSFDI vs. *ex vivo* analysis.

To support our eSFDI quantification results, we performed *ex vivo* measurements with samples taken from the imaged sites of *in vivo* drug release in mice. As Fig. 7A shows, tumor tissue from mice that received both drug and light treatment had an average Dox concentration of 5.6 ± 0.8 µg/g, while peripheral muscle had 2.5 ± 0.7 µg/g and contralateral muscle tissue that did not receive light had 1.3 ± 0.2 µg/g. Tissue removed from the control mouse that received drug but no light had 1.2 ± 0.1 µg/g Dox concentration across all tissue sites, while tissue removed from mice with no injected LC-Dox-PoP showed only a small signal due to background autofluorescence (0.04 ± 0.07 µg/g).

As Fig. 7B shows, the Pearson correlation coefficient between the average Dox quantified with eSFDI to the average Dox quantified with *ex vivo* analysis was 0.77 with a p value of 0.0011, indicating that there was a clear linear relationship between the two methods. A direct one-to-one relationship was difficult to test, since the true density of the tissue samples was unknown but only their mass was known. The extracted Dox concentration from the contralateral muscle of the treated group (1.3 ± 0.2 µg/g) matched well with the average of all three samples from the control mouse injected with LC-Dox-PoP but not illuminated (1.2 ± 0.1 µg/g), further demonstrating that the release occurred only at the sites of light illumination.

## Discussion

In this study, we have introduced a quantitative endoscopic imaging approach that used dual-channel image fibers for patterned illumination and detection in both reflectance and fluorescence imaging modes, to quantify optical and fluorescence parameters, respectively. Endoscopic SFDI (eSFDI)-quantified optical parameters provided *a priori* information for a correction factor to obtain accurate drug fluorescence concentrations. Thus, this approach allowed quantification of absolute Dox fluorescence concentration by compensating for variations in fluorescence signal due to absorption and scattering at both excitation and emission wavelengths^40,43,49^. Quantitative eSFDI provided distributions of locally released (bioavailable) absolute Dox concentration, which could provide a feedback for the duration of treatment to be adjusted to ensure full drug release from the liposomes.

Others have previously shown the use of rigid endoscope approach for optical property quantification^46,47^. Angelo *et al*. utilized a dual channel rigid endoscope to project patterns and acquire reflectance images^46,48^. Their system utilized single snapshot of optical property (SSOP) imaging to provide real-time images of optical properties within 5% agreement of standard SFDI images. Our flexible fiber endoscope system had slightly lower accuracy for quantification of optical properties (~15% error for high absorption and scattering calibration phantom). Using plastic flexible image fibers might decrease the accuracy of the optical property quantification. We also used traditional SFDI approach, which involves three-phase and multi-frequency projection and thus it is slower than snapshot method. Implementing snapshot acquisition to fiber-based eSFDI is expected to be straightforward.

We utilized the fluorescence correction method of Gardner *et al*.^55^ to quantify Dox concentration. Previous work has shown that this method works well with the SFDI approach and produce accurate results^40,41,43^. Our results indicate that we have ~15% error in quantification of Dox fluorescence concentration. Any increased error in optical property quantification likely propagated to the fluorescence quantification, which requires the absorption and scattering parameters at the excitation and emission wavelengths as inputs. Alternatively, we could have used the fluorescence correction model developed by Kim *et al*.^38^ which utilized the absorption coefficient at the excitation wavelength along with the reflectance at the excitation and emission wavelength to correct the raw fluorescence signal, as was recently demonstrated in a widefield imaging system^56^. In future studies, we will investigate implementing higher quality image fiber and the effects of different fluorescence quantification models to improve quantification of optical properties and drug concentrations.

The reflectance images showed substantial variations with respect to the raw counts and surface geometries. Yet we were still able to resolve the quantified drug release within an expected range coinciding with the phantom calibration results. Thus, proposed approach demonstrates the effectiveness of these methods of correcting for optical properties and height variations. For the fluorescence images, it is interesting to note that by utilizing raw fluorescence signal alone (Fig. 5A) one could wrongly infer that the release of bioavailable Dox was still incomplete by 8 min, whereas the quantified Dox concentration release (Fig. 5B) indicates that 8 min of treatment light irradiation was enough for both phantoms to have complete release. These results imply that, in certain cases, quantification of the released (bioavailable) Dox concentration could be crucial for accurately treating tumors for individualized optimization.

The light dose was delivered uniformly across the treatment area and the fluence rate was chosen based on previous mouse studies. In areas with very heterogeneous optical properties, a uniform light dose might not be the best delivery option. Knowledge of the tissue optical properties and liposome distribution before light delivery, as well as the changes caused by triggered release, would allow for optimization on an individual, tumor-by-tumor basis. Our eSFDI setup is well equipped to perform these measurements and, in future studies we will integrate the treatment laser with the Digital Micromirror Device (DMD) to optimize the shape and intensity of the treatment light on an individual basis.

The light delivery was centered on the tumor, which minimized the release in surrounding muscle and contralateral muscle. The targeted release ensured that minimal Dox was released in healthy tissue. This is important to minimize the adverse effects common with systemic chemotherapy. In this study, the tumor was directly beneath the surface and therefore light could easily be directed to the desired area and excluded from others. However, ovarian micromets appear in the peritoneal cavity, where wide-field light delivery is possible under surgical openings. Alternative modes of light delivery, such as interstitial fiber-optics and endoscopes, will also work for drug release. 3D fluorescence imaging would also provide a more accurate approach for reducing or eliminating partial volume effects such as those originating from layers *in vivo* ^53,57^.

There exist several more opportunities to improve our work. We used OVCAR-3 tumor cell injection subcutaneously to obtain superficial tumors to demonstrate the capability of our endoscopic imaging. More clinically-relevant approach would be intraperitoneal injection to obtain micromets that would mimic human disseminated ovarian carcinoma^5,6^. In this respect, our own experience has proven that mouse model with OVCAR-3 cell line is challenging, and currently we are working on a NuTu-19 cell line and Fischer 344 rat model that would allow reproducible and efficient way of studying ovarian peritoneal carcinomatosis, as well as studying invasive procedures like surgery, fiber based imaging and light delivery^58,59^. Moreover, our endoscope provides mesoscopic scale imaging; it has the advantage of providing mm- to cm-scale imaging field and ~100-micron resolution. Thus, we expect the endoscope would be advantageous for quick screening of suspicious regions and for detecting of small nodules that consist of thousands of cells, but would not detect or resolve individual cells^5,6^. A combination with microendoscopy might allow multi-scale (micron and sub-millimeter resolution) imaging, so that our “mesoscopic endoscope” would define the suspicious imaging field rapidly and microendoscopy would provide the “zoomed-in” version of the suspicious field with cellular resolution^6^. Furthermore, we mainly focused on small sizes of tumors (~3 mm diameter, thickness is even smaller due to flat nature of development in their very early stage) to mimic micromets. We did not correct for the surface angle variations since these tumor size and height were small. For larger tumors or applications with larger surface angles, we would need to implement a model-based Lambertian reflectance approach for angle correction, as demonstrated by Gioux *et al*.^60^. Finally, current analysis requires post-processing that takes about 5 minutes depending on the pixel number and binning. Implementing a GPU-based fast Monte Carlo model would allow mapping of the released drug concentrations in near real time^31,61^. Fast mapping of drug concentrations would provide time window for image-based feedback to optimize drug delivery.

In conclusion, we have developed and validated a novel eSFDI system with the capability to quantify the absolute concentration of Dox in phantoms and in animals. We showed that eSFDI could accurately quantify Dox concentration distribution noninvasively. Noninvasive, fast optical techniques can assess bioavailable drug content *in vivo*, and thereby therapeutic activity and response. Ultimately, this will lead to optimization and control of drug release with both higher response rates and many fewer side effects. This study demonstrates the localized NIR-light triggering based photoactivation of Dox with minimally invasive, quantitative measures. The ability to characterize drug release makes eSFDI a potentially useful tool for evaluating and characterizing novel drug release and delivery *in vivo* for fast clinical translation.

## Materials and Methods

### Custom endoscopic SFDI imaging (eSFDI) and release setup

**Figure 8:**
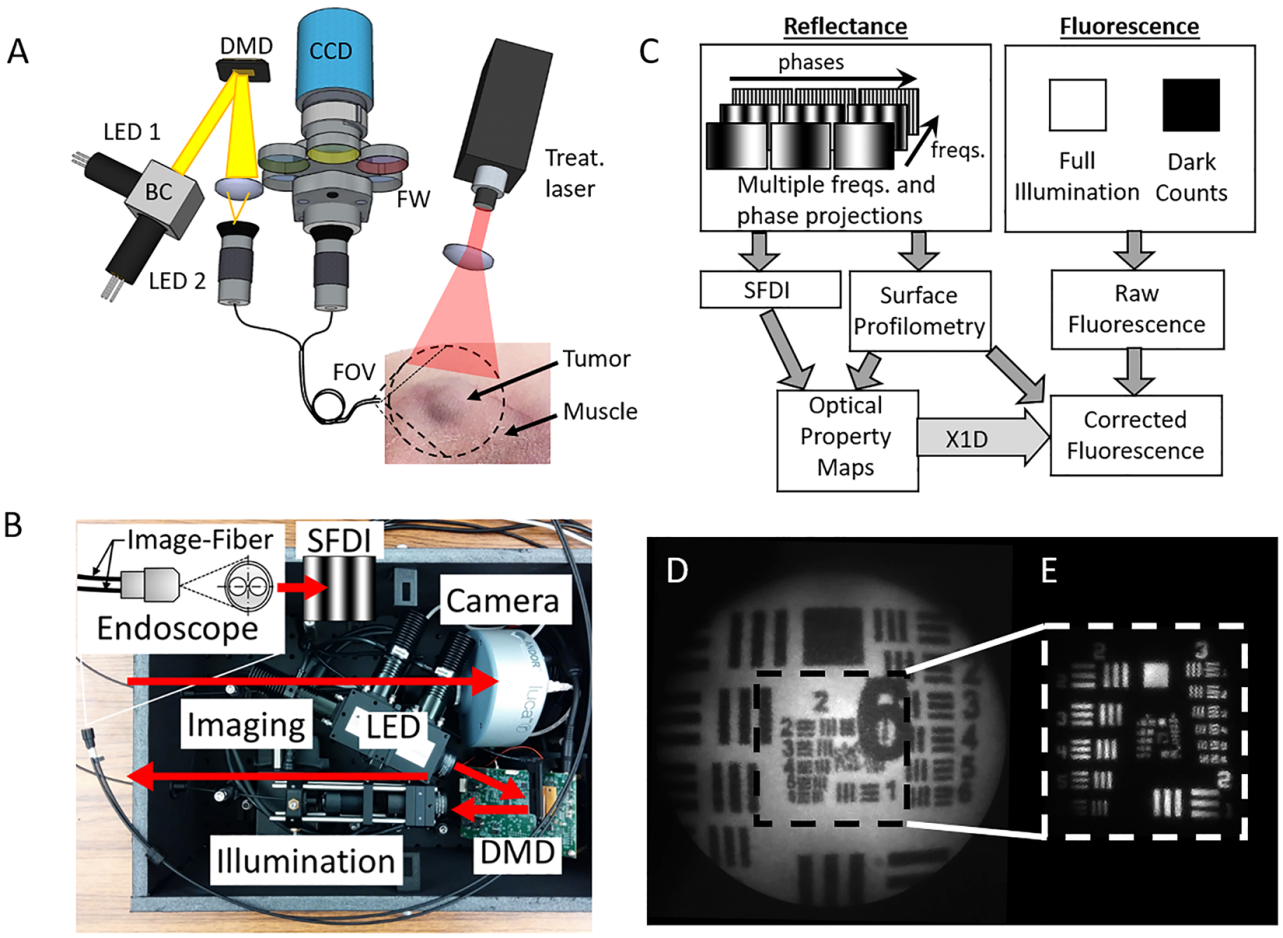
System design and characterization. (**A**) Dual-modal, dual-channel endoscope that allows combined imaging and treatment scheme in a single platform. Structured light is projected onto tumor using the custom endoscope. Mouse flank shown highlighting the tumor and surrounding muscle. (**B**) Picture of compact and portable version of the instrument highlighting the separate components. (**C**) Overview of the system operating in two imaging modes (reflectance and fluorescence) to obtain corrected fluorescence maps for quantification of true Dox concentration. (**D**) Endoscopic image of fluorescent-positive United States Air Force (USAF) target. (**E**) Reflectance image of negative USAF target. Outlined region in (**D**) provides relative scale of the Fluorescence and Reflectance modalities.

Figure 8A shows the diagram of the instrument with the dual-channel fiber endoscope design and Fig. 8B shows the internal layout of the system. The instrument was custom built, utilizing two flexible plastic image fibers. This dual-channel approach allowed light projection and detection imaging to be performed in separate, dedicated channels. The objective lenses in the tips of the fiberscope indicate the overlapping field of views (FOVs) of illumination and detection. Two compact light-emitting diodes (LEDs), both LCS series from Mightex, CA, with central wavelengths of 490 nm and 590 nm were used for measuring Dox excitation/fluorescence (490/590 nm) in the LC-Dox-PoP nanoconstruct. The 490 nm LED excitation light was bandpass-filtered at 490 nm ± 20 nm for imaging Dox optical properties at the excitation peak, and a 590 nm ± 20 nm band-pass filter was used to quantify optical properties at the emission wavelength of Dox. An LED controller (Mightex, CA) sequentially selected the desired excitation wavelength and light was projected though a compact Digital Micromirror Device (DMD) module (LightCrafter 4500, Texas Instruments), where it is spatially modulated to the desired frequency and phase pattern. The DMD module generated the appropriate sine wave patterns with three different phases (0, 2π/3, 4π/3) and four spatial frequencies from 0 to 2.0 cm^−1^. The patterns were focused into the image fiber and relayed onto the surface where reflected light was collected with the second fiber and relayed to the EMCCD camera (1004 × 1002 pixels, Luca, Andor, Belfast, Ireland). The EMCCD camera is compact yet highly sensitive, with an EM gain for imaging at low signal levels of fluorescence contrast. The camera was focused on the same field of view (FOV) as the projected pattern (1.6 cm × 1.6 cm). The CCD acquisition time was set to 0.1 s in reflectance mode for a total SFDI acquisition time of ~2.4 seconds to acquire all images at both wavelengths. For fluorescence mode, a single 2 s acquisition was performed with excitation light on followed by a 2 second dark image with excitation light off for a total time of 4 seconds. Cross-polarizers were built into the front of the endoscope’s distal end to reject specular reflection. Custom objective lenses (diameter: 1 mm, angular FOV: 70°) at the distal end of the instrument ensured that the projection FOV matched the imaging FOV. A structural frame acted as a mechanical stop to ensure that the object surface was no closer than the minimal effective working distance of the scope. Due to the divergence of light from the objective lenses, the frequency of the projected SFDI patterns will increase with distance, so the structural frame ensured that the target was always close to the optimal working distance. The reflected and fluorescence light collected by the imaging fiber was relayed and focused through a dichroic mirror onto the camera. The image fiber contained 30,000 picture elements in a 650 µm diameter bundle (high resolution, low power – Myriad Fiber Imaging). The imaging fibers are selectable with respect to desired resolution and signal to noise ratio (SNR). For *in vivo* measurements, we used a 2000 µm diameter image circle with lower resolution (13,000 image elements) to obtain high photon counts. For the release experiments, the treatment light was a 657 nm laser with a fluence rate of 350 mW/cm^2^ and the diameter of the beam was adjusted for each application. The entire system was automated by a custom MATLAB software program and an overview of the workflow is shown in Fig. 8C.

### Optical System Characterization

The performance of the endoscopic imaging system was first evaluated by imaging fluorescent and reflective USAF resolution targets (R1DS1N, Thorlabs) to determine the imaging capability of the smallest resolvable line pair spacing and thereby the highest spatial resolution of the system. The image fiber output was expanded to the full resolution of the CCD, which corresponded to approximately 0.2 elements per pixel for the 30,000-element fiber. A single image acquisition time was mainly dependent on the CCD image acquisition in fluorescence mode and how many spatial frequencies were used. Typical performance times were as follows: In reflectance mode, 100 ms per image, 3 phase, and 2 frequency, the total acquisition time was approximately 0.6 s. In fluorescence mode, only DC fluorescence image at 1.0 s was sufficient to obtain DOX fluorescence *in vivo*. Frequency filtering was performed to remove honeycomb noise from the fiber bundle. Distortion correction was applied to remove warping in the test target. At a 1.6 × 1.6 cm FOV, the system could resolve group 2, element 5 of the target, corresponding to a line width of 78.75 µm, which is equivalent to 6.35 line pairs/mm as shown in Fig. 8D. Figure 8E demonstrates a higher magnification (with a smaller FOV of 0.8 cm × 0.8 cm), at which the endoscopic system can resolve group 3, element 5 of the target, corresponding to line width 39.37 µm, which is equivalent to 12.7 line pairs/mm.

### Mouse model of ovarian cancer

All animal experiments followed protocols approved by the IACUC of Wright State University (WSU). All animal care was performed in accordance with the relevant guidelines and regulations outlined in the “Guide for Care and Use of Laboratory Animals”.

Nine female nude mice were inoculated subcutaneously with 10^7^ OVCAR3 cells. Only females were used, as males do not contract this disease. When tumors reached approximately 3 mm in diameter, mice were injected i.v. with 50 µg/mL LC-Dox-PoP liposomes by body weight, for a final dose of 5 mg/kg; and imaged 1 hour later. For the imaging, mice were placed on a heating pad and anesthetized with Isoflurane. The mouse was raised into contact with the structural frame and positioned so the tumor was centered in the field of view of the scope. eSFDI measurements were acquired and then NIR treatment light (657 nm, 300 mW/cm^2^, 1.6 cm diameter) was directed onto the tumor to release the Dox. Dox fluorescence was measured continuously during release. Fluorescence measurements were acquired every 10 seconds to assess the release dynamics during treatment. In total, seven mice received LC-Dox-PoP + treatment light, one mouse received the injection of LC-Dox-PoP, but no treatment light and one mouse received treatment light without any injected LC-Dox-PoP. After release, mice were euthanized and tissue samples from the tumor, surrounding muscle, and contralateral muscle tissues were removed.

### Optical Property Quantification using Reflectance Data

Bulk optical absorption (µ_a_) and scattering (µ’_s_) parameters were quantified by fitting spatial frequency domain reflectance (*R*_*d*_(*f*)) data with a modified frequency-domain Monte Carlo model by using a reference phantom with known optical properties and a custom Matlab program with the lsqcurvefit nonlinear fitting algorithm^42^. Two different initial conditions were used to check for possible local minima. To quantify absorption and scattering parameters, 4 spatial frequencies from 0 to 2.0 cm^−1^ and three phases (0, 2π/3, 4π/3) were used.

### Drug Concentration using Attenuation-Compensated Fluorescence Analysis

The quantification of Dox fluorescence concentration was performed by using the Gardner model. This corrects raw fluorescence signal by compensating for optical absorption (µ_a_) and scattering (µ’_s_) loss, both at excitation and emission wavelengths^40,43,55^. In this model, the fluorescence correction factor, *X*_1*D*_(*λ*_*ex*_, *λ*_*em*_), is determined, where *X*_1*D*_(*λ*_*ex*_, *λ*_*em*_) represents the effective path-length during excitation light penetration and escape of emitted fluorescence from the tissue. Then we calculated the corrected fluorescence as *F*_*car*_ = *F*_*caw*_/*X*_1*D*_, where *F*_*caw*_ is the measured raw signal, *X*_1*D*_ is the correction factor and *F*_*corr*_ is the corrected fluorescence by compensating the optical absorption, scattering, and light propagation. By using the calibration factor, transition to Dox concentrations was established.

### Correction for Geometrical Effects

For the *in vivo* measurements, it was necessary to correct for the effect of surface height variation to improve quantification accuracy of optical parameters and drug release pharmacokinetics. While the structural frame prevented the mouse from being closer than the working distance of the scope, it was still possible for tissue surrounding the tumor to be lower than the focal plane, therefore affecting the intensity of the collected reflectance and fluorescence light. To obtain the height-corrected tissue optical properties and corrected fluorescence emission, we implemented a phase-shifting profilometry approach^60,62^ to correct for the effect of height differences. Briefly, we first acquired measurements of a flat phantom with known optical properties from fixed heights below the frame [0, 2, 4, 6, and 8 mm]. The maximum distance of 8 mm was chosen because that was the range of expected height variations for the *in vivo* experiments. For other applications where the working distance may vary more, the process could be repeated again to cover the expected range. The phase image^63^ was obtained for the highest spatial frequency (2.0 cm^−1^) and the corresponding modulation amplitude (*M*_AC_) image was obtained at each height. We then applied a linear fit at each height (fitting phase versus *M*_AC_). Next, we used the corresponding height map to retrieve the height-adjusted reference *M*_AC_, which was then used to compute the corrected diffuse reflectance (*R*_d.correct_). Finally, eSFDI optical property quantification was performed after obtaining the *R*_d.correct_.

### Long-circulating Dox in PoP Liposomes (LC-Dox-PoP)

The PoP used in this study was a porphyrin-phospholipid, which was generated as previously described^64^. The LC-Dox-PoP was formulated with ethanol injection, extrusion, and active drug loading as previously described^30^. PoP liposomes consisted of 53 mol. % 1,2-distearoyl-sn-glycero-3-phosphocholine (DSPC, Cat#LP-R4-076), 5 mol. % 1,2-distearoyl-sn-glycero-3 phosphoethanolamine-N-[methoxy(polyethylene glycol)-2000] (DSPE-PEG-2K, Cat# LP-R4-039), 40 mol. % cholesterol (Cat# CH-0355), and 2 mol. % Pyro-lipid. Liposomes were prepared using a modified ethanol injection method. Lipids (200 mg total) were dissolved in 2 mL ethanol heated to 60 °C and mixed with 8 mL 250 mM ammonia sulfate (pH 5.5). This solution was then extruded using a 10 mL Lipex extruder with stacked 200 nm, 100 nm, 80 nm membranes. Following extrusion, the liposomes buffer exchange was achieved by dialysis using 12,000-14,000 MW cut off tubing (Cat # D/N OD 100C34) and a 10 mM HEPES 10% Sucrose buffer. Doxorubicin was loaded into the liposomes by adding Doxorubicin and incubating at 60 °C for 1 hour.

Liposome sizes and polydispersity were determined by dynamic light scattering via NanoBrook 90 Plus PALS in phosphate-buffered saline (Cat#14190_144). Loading efficiency was determined by running 25 mL of liposomes (~20 mg/ml lipids) diluted in 1 mL PBS over a Sephadex G-75 column. 24 × 1 mL fractions were collected and the loading efficiency was determined as the percentage of the drugs in the liposome-containing fractions (the first 4–8 mL). Dox was measured by fluorescence (excitation/emission: 480 nm/590 nm) using a TECAN Safire fluorescent microplate reader. Emission spectrums for Dox-PoP were measured using a PTI fluorimeter. PoP liposomes (1 mg/mL Dox) were diluted 200 times in PBS and the Porphyrin and Dox emission was measured at 420 nm and 480 nm respectively. 0.25% Triton X-100 was added to lyse liposomes, and emission were measured.

### *Ex vivo* Dox analysis

*Ex vivo* gross quantification of Dox in tissue samples was performed on extracted tissue samples. After light treatment, the mice were euthanized and tissue samples removed. The tumor and surrounding muscle samples were obtained from tissue sites illuminated with treatment light; also, additional muscle samples were taken from the contralateral side not illuminated with treatment light, to serve as a control. Each sample was placed in nuclear lysis buffer [0.25 M sucrose, 5 mM Tris-HCl, 1 mM MgSO_4_, 1 mM CaCl_2_ (pH 7.6)] and homogenized before being stored at −80C. Once all mice were treated, all samples were removed and processed at the same time. 100 µL of homogenate was removed and added to 900 µL of extraction buffer (0.075 N HCl 90% isopropanol) and stored overnight at −20 °C. After thawing, the vials were centrifuged and 200 µL samples of liquid were drawn and added to separate wells of a 96-well plate. Three samples from each tissue type were used to obtain average values. A standard curve of known Dox concentration was measured in the same plate to obtain the conversion curve between fluorescence counts to Dox concentration. The 96-well plate was read with a Synergy H1 micro plate reader (BioTek, Winooski, VT). For each well, the excitation wavelength was 480 nm and the emission spectra was between 530 nm and 700 nm with 10 nm step size resolution. Background counts were acquired by obtaining the measurements from a well that had only excitation buffer. The background-subtracted raw fluorescence spectra were fit to a model that included Dox- and autofluorescence-basis spectra.

### Data availability

The datasets generated and analyzed during the current study are available from the corresponding author upon reasonable request.
